# A Comprehensive Analysis of the Myocardial Transcriptome in *ZBED6*-Knockout Bama Xiang Pigs

**DOI:** 10.3390/genes13081382

**Published:** 2022-08-01

**Authors:** Shengnan Wang, Wenjie Tian, Dengke Pan, Ling Liu, Cheng Xu, Yuehui Ma, Dandan Wang, Lin Jiang

**Affiliations:** 1 Laboratory of Animal (Poultry) Genetics Breeding and Reproduction, Ministry of Agriculture, Institute of Animal Science, Chinese Academy of Agricultural Sciences (CAAS), Beijing 100193, China; namowsn@hotmail.com (S.W.); xuchengcaas@163.com (C.X.); yuehui.ma@263.net (Y.M.); 2 National Germplasm Center of Domestic Animal Resources, Ministry of Science and Technology of the People’s Republic of China, Institute of Animal Science, Chinese Academy of Agricultural Sciences (CAAS), Beijing 100193, China; twj1938016551@163.com (W.T.); liuulingg602@163.com (L.L.); 3 State Key Laboratory for Conservation and Utilization of Subtropical Agro-Bioresources, College of Animal Science and Technology, Guangxi University, Nanning 530004, China; 4 Institute of Organ Transplantation, Sichuan Academy of Medical Sciences & Sichuan Provincial People’s Hospital, Chengdu 610072, China; pandengke2002@163.com; 5 College of Animal Science and Technology, Qingdao Agricultural University, Qingdao 266109, China

**Keywords:** *ZBED6*, lncRNA, heart, RNA-seq, Bama Xiang pig

## Abstract

The *ZBED6* gene is a transcription factor that regulates the expression of *IGF2* and affects muscle growth and development. However, its effect on the growth and development of the heart is still unknown. Emerging evidence suggests that long noncoding RNAs (lncRNAs) can regulate genes at the epigenetic, transcriptional, and posttranscriptional levels and play an important role in the development of eukaryotes. To investigate the function of *ZBED6* in the cardiac development of pigs, we constructed the expression profiles of mRNAs and lncRNAs in myocardial tissue obtained from Bama Xiang pigs in the *ZBED6* knockout group (*ZBED6*-KO) and the wild-type group (*ZBED6*-WT). A total of 248 differentially expressed genes (DEGs) and 209 differentially expressed lncRNAs (DELs) were detected, and 105 potential cis target genes of DELs were identified. The functional annotation analysis based on the Gene Ontology (GO) and Kyoto Encyclopaedia of Genes and Genomes (KEGG) databases revealed two GO items related to muscle development by the cis target genes of DELs. Moreover, *IGF2* was the direct target gene of *ZBED6* by ChIP-PCR experiment. Our results explored the mechanism and expression profile of mRNAs and lncRNAs of *ZBED6* gene knockout on myocardium tissue development, mining the key candidate genes in that process like *IGF2*.

## 1. Introduction

Pork is the third primary source of animal protein for human consumption and is still the most important meat production and marketing variety in the world (FAO). China is one of the leading countries in pork consumption. According to FAO statistics, China’s pork production ranked first in the world in 2020. Pork plays an important role in China’s consumption and daily diet and a vital role in the national economy. With the development of society and improvements in people’s economic levels, consumers are paying more attention to the quality of pork. The world’s average supply of protein of animal origin (g/cap/day) has increased from 26.8 g in 2000 to 31.6 g today (FAO). At present, the fat content of pork in China is high, but the amount of muscle is insufficient. Certain problems have been found in Chinese pork breeds, such as a low lean meat rate and a lack of lean pig breeds to conduct breed improvements [1]. Moreover, domestic pig breeding started relatively late, and most of the core provenances of lean pigs rely on imports. Therefore, continuous improvement of the quality of pork has become a key problem in breeding.

The Bama Xiang pig is a breed that has been bred in China for more than 20 years, with good genetic consistency and stability [2], and its organ characteristics, proportions and most physiological and biochemical indices are very similar to those of humans [3]. Therefore, Bama Xiang pigs are regarded as an important large animal research model in life science.

*ZBED6* (zinc finger, BED-type containing 6) is a transcription factor in mammals that can combine with *IGF2* (insulin-like growth factor 2) to upregulate its expression level, thus reducing the deposition of subcutaneous fat [4]. This transcription factor has been shown to be closely related to the growth and development of muscles [5], and it can inhibit the formation of myotubes during the process of cell differentiation at the cellular level [6]. In cattle, *ZBED6* can promote the differentiation of myoblasts by inhibiting the activity of *IGF2* [7]. In mouse C2C12 cells, it was found that ZBED6 downregulated the expression of *Myod1* and *Mef2c* genes and thus regulated the formation of muscular tubes during cellular differentiation [6].

To study the regulatory mechanism of the ZBED6-IGF2 axis on muscle growth and development, Younis et al. constructed *ZBED6* knockout mice and *IGF2* mutant mice to upregulate *IGF2* expression [4]. They found that *ZBED6* knockout mice and *IGF2* mutant mice had larger hearts, and there was a significant difference between female wild-type and *IGF2* mutant mice. In addition, *ZBED6* knockout and *IGF2* mutation had no superimposed effect on the function and had the same effect on the heart. In recent years, with the development and maturity of high-throughput sequencing technology and CRISPR/Cas9 gene editing technology, it has become possible to use transgenic pigs as a model to study the molecular function of *ZBED6* [8,9,10].

LncRNAs were once considered “noise” in gene expression products and were thought to have no biological function [11], which has been proved wrong. With the development of sequencing technology and bioinformatics, an increasing number of lncRNAs have been found to play an important role in the regulation of the cell cycle [12], epigenetics [13], disease occurrence [14] and many important biological processes [15,16]. At present, lncRNAs related to reproductive traits [17,18], growth traits [19,20], meat quality traits [21,22,23], and pig diseases [24] have been reported. We have already studied the lncRNAs involved in skeletal muscle development [25]; however, the specific function of lncRNAs in the heart after *ZBED6* knockout is still unknown.

In our previous research, the significantly DEGs (differentially expressed genes) were enriched in signals of muscle development and the immune response between the *ZBED6*-KO (*ZBED6* knockout) Bama Xiang pigs and the *ZBED6*-WT (wild-type) group. This indicated that *ZBED6* was likely to affect the related functions of heart tissue [26]. Although our early study provided clues for the *ZBED6* gene in the heart development of domestic pigs, how lncRNAs affect heart development and the underlying regulatory mechanism in *ZBED6*-KO pigs remain unclear. Therefore, it is of scientific interest to analyze why the knockout of *ZBED6* leads to an increase in heart weight and the specific mechanism underlying myocardial development.

In this study, the roles of mRNAs and lncRNAs in myocardial growth and development were investigated at the transcriptome level based on comparative transcriptome analysis using *ZBED6*-KO Bama Xiang pigs that were constructed successfully in the previous stage. The results provide new clues for the function of *ZBED6* and contribute to the further improvement and breeding of domestic pigs.

## 2. Materials and Methods

### 2.1. Laboratory Animals

*ZBED6* gene knockout Bama Xiang pigs (*ZBED6*-KO) and normal wild-type Bama Xiang pigs (*ZBED6*-WT) of the same age were provided by the pig farm affiliated with the Institute of Animal Science, Chinese Academy of Agricultural Sciences (CAAS), Beijing, China. The pigs had ad libitum access to a commercial pig diet and water throughout the experimental period. The knockout of the *ZBED6* gene was completed by the Pan Dengke Research Group of the Institute of Animal Sciences of the Chinese Academy of Agricultural Sciences, Beijing, China, using CRISPR/Cas9 technology.

### 2.2. Sample Collection and Total RNA Extraction

Heart tissues were collected from three 8-month-old *ZBED6*-KO pigs (×1, ×3, and ×6) and three *ZBED6*-WT pigs (×2, ×4, and ×5) of the same age and then preserved in RNA later reagent (Thermo Fisher Scientific, Waltham, MA, USA) for RNA extraction (Supplementary Figure S1A).

Total RNA for RNA sequencing (RNA-seq) was isolated from six myocardial samples taken from the interventricular septum muscle (three from each *ZBED6*-WT and *ZBED6*-KO pigs) with an RNeasy Mini kit (QIAGEN, Dusseldorf, Germany) according to the operation manual of the kit for specific operation steps. One microgram of RNA from each sample was reverse transcribed with the RT-PCR kit of TaKaRa Company (TaKaRa, Dalian, China). The purity and concentration of the RNA samples were evaluated on a NanoDrop 1000 spectrophotometer (Thermo Scientific, Waltham, MA, USA), and standard denaturing agarose gel electrophoresis was used to monitor the degradation and contamination. An Agilent 2100 bioanalyzer system with an RNA Nano 6000 Assay Kit (Agilent Technologies, Palo Alto, CA, USA) was used to verify the quality and quantity of RNA samples. Samples with a concentration greater than 100 ng/μL, a total amount equal to or greater than 2.0 μg and an RIN value greater than 8.0 were used for the RNA-seq analysis.

### 2.3. Library Construction and Sequencing

According to the quality of the RNA, a total of 6 samples were sequenced. Using NEBNext Poly (A) mRNA Magnetic Isolation Module to separate poly(A) mRNA from total RNA. NEBNext^®^ Ultra™ RNA Library Prep Kit (Illumina Inc., San Diego, CA, USA) was used to prepare the Sequencing library of 2 µg total RNA, the kit produced a 275 bp fragment including the average size of the adapters. In accordance with the instructions for the TruSeq PE Cluster Kit v3-cBot-HS kit (PE-401-3001, Illumina Inc., San Diego, CA, USA), the cBot Cluster Generation system was used to cluster samples with index joints. After the cluster was formed, the above samples were sequenced on the Illumina Hiseq 2500 platform for 150 cycles in paired-end mode, and 150 bp paired-end reads were generated. The Fastq files were uploaded on the Sequence Read Archive database: https://www.ncbi.nlm.nih.gov/sra, accessed on 1 June 2021 with the accession number PRJNA663759.

### 2.4. RNA-Seq Analysis

The raw data in Fastq format were first processed by inhouse scripts. The original reads of Illumina sequencing were obtained by removing the reads containing adapters, the reads containing ployN and the low-quality reads from the original data. FastQC v0.11.4 software was used to filter the original reads, and the low-quality bases (Phred value < 20) at the end of the reads and the reads with lengths less than 25 bp were removed. The Q20, Q30 and GC contents of clean reads were calculated. HISAT2 (v2.0.4 by Daehwan Kim) software [27] with default parameters was used to compare clean data after quality control with the Ensemble Sscrofa11.1 reference genome: ftp://ftp.ensembl.org/pub/release-101/fasta/sus_scrofa/DNA/Sus_scrofa.Sscrofa11.1.DNA.toplevel.fa.gz, accessed on 10 April 2022. StringTie software (v1.3.4d) carried out the reconstruction of transcripts. Together with HISAT2, it allows biologists to identify new genes and new spice variants of known ones [28,29].

### 2.5. Identification of lncRNAs

All the transcripts were merged using Cuffmerge (v2.2.1) [30] software. First, assembled transcripts shorter than 200 bp and 2 exons were filtered. Then, we removed the “Annotated_mRNA” and “Annotated _lncRNA” in Ensemble Sscrofa11.1 reference genome’s gtf. We utilized CPC2 [31], CNCI [32], Pfam [33] and PLEK [34] with default parameters to predict novel transcripts with coding potential. The characteristics of lncRNA were compared with mRNA.

### 2.6. Quantification of mRNA and lncRNA Expression

The quantification of the transcripts and genes was performed using RSEM [35] software, and reads per kilobase of transcript per million mapped reads (RPKM) were obtained. The EdgeR [36] R package was used for differential expression analysis.

The differentially expressed genes (DEGs) were scanned with *p*-value ≤ 0.05, fold change (FC) ≥ 1.5 and value of each sample ≥1. The differentially expressed lncRNAs (DELs) between *ZBED6*-KO group and *ZBED6*-WT group were identified with *p*-value ≤ 0.05, fold change (FC) ≥ 1.5 and value of each sample ≥0. The pipeline used to find DEGs and DELs between two groups from the RNA-seq data is shown in Supplementary Figure S1B.

### 2.7. lncRNA Target Genes Prediction

Among its roles, lncRNA can regulate the expression of mRNA by cis or trans. When the role of lncRNA is limited to the transcriptional chromosomes (especially neighboring genes), it is mainly regulated by cis, and when it affects the expression of genes on other chromosomes (remote), it plays the role of trans [37]. Based on the theory of the cis-acting regulatory element, the protein coding genes located within 100 kb from lncRNA were selected as potential cis-acting target; for trans-acting target prediction, the correlation coefficients between the coding genes and lncRNAs were calculated, which required the sample size to be more than five. Therefore, we did not predict the trans target genes of DELs in this study.

### 2.8. Cluster Analysis

The RPKM values for all of the annotated transcripts from the six samples transcriptomes were used to perform PCA, which was implemented with the “ggfortify” package in R (version 3.1.3, http://cran.r-project.org/, accessed on 18 April 2022). The hierarchical cluster analysis of DEGs was carried out using the cluster analysis function in the “pheatmap” package of R language.

### 2.9. Enrichment Analysis

Gene Ontology and Kyoto Encyclopedia of Genes and Genomes pathway Analyses of DEGs and DELs were carried out with the g:Profiler web server: http://biit.cs.ut.ee/gprofiler/, accessed on 23 April 2022 [38]. Significantly enriched GO terms or KEGG pathways were identified by Benjamini-Hochberg FDR, and *p*.adj < 0.05 was used as the threshold. The results were visualized using the R language package.

### 2.10. Chromatin Immunoprecipitation (ChIP)-PCR Analysis

ChIP was performed in native conditions. Briefly, freshly snap-frozen tissues were treated with 1% formaldehyde in medium for 18 min and neutralized with glycine (AMRESCO, Radnor, PA, USA) for 5 min at room temperature. The tissues were smashed and resuspended in SDS lysis buffer (Beyotime, Shanghai, China). After incubation for 30 min at 4 °C, the lysates were sonicated 24 times (30 s each) (Bioruptor^®^ Sonication System, Denville, NJ, USA). An equal amount of chromatin was immunoprecipitated at 4 °C overnight with 8 ug of the following antibodies: anti-ZBED6 (Atlas Antibodies, Stockholm, Sweden, HPA068807) and normal mouse IgG (CST, 2729S). Immunoprecipitate were collected after incubation with Protein G agarose beads (ThermoFisher Scientific, Waltham, MA, USA). The beads were washed, and bound chromatin was eluted in ChIP Elution Buffer. Then, 20 microliters 5 M NaCl was added to the combined eluates and reverse histone-DNA crosslinks by heating at 65 °C for 4 h, after which RNase A (TIANGEN, Beijing, China) was added for 30 min at 37 °C; then the proteins were digested with Proteinase K (TIANGEN, Beijing, China) for 2 h at 65 °C. The coprecipitated DNAs were purified using a ChIP DNA Clean & Concentrator purification spin column (ZYMO, Irvine, CA, USA) and eluted in 20 μL dilution buffer. The *ZBED6* binding site was evaluated using PCR and normalized with total chromatin (input). Normal mouse IgG was used as the negative control, and the primers are described in Supplementary Table S1. The PCR conditions were: 1 cycle at 95 °C for 5 min followed by 32 cycles at 95 °C for 30 s, Tm °C for 30 s, and 72 °C for 30 s. The PCR products were then electrophoresed on 1.5% agarose gels stained with GelGreen (TIANGEN, Beijing, China).

It has been shown that *ZBED6* can bind to *IGF2* through the “GCTCG” motif and then inhibit its expression in mammals [4,5]. Previous ChIP-seq using mouse C2C12 cells showed that the *ZBED6* binding site of *IGF2* corresponding to the porcine QTN site was among the most highly enriched regions [5]. Therefore, we blasted the *IGF2* binding sequence in C2C12 with *Sus scrofa IGF2* located on chromosome 2 and designed primers in the blast region for ChIP-PCR to examine the binding of *IGF2* and *ZBED6.*

### 2.11. Quantitative Real-Time PCR (RT-qPCR) Analysis

Differentially expressed genes were randomly selected to identify the gene expression. In order to verify the stability of candidate genes in offspring, myocardial tissue samples of six three-month-old individuals of F3 generation pigs were collected, three in each group.

For the RT-qPCR analysis of genes, reverse transcription was performed using a PrimerScriptTM RT regent Kit (TaKaRa, Dalian, China) according to the manufacturer’s instructions. RT-qPCR with TB Green^®^ Premix Ex Taq™ II (TaKaRa, Dalian, China) was performed with a QuantStudio^TM^ 7 Flex 96-well System (Thermo Fisher Scientific, Waltham, MA, USA) as follows: 95 °C for 30 s; 40 cycles of 95 °C for 5 s, 60 °C for 34 s; and 95 °C for 15 s, 60 °C for 1 min, and 95 °C for 15 s. The data were analyzed with the 2^−ΔΔCt^ method [39] (Livak & Schmittgen 2001). Relative gene expression data were analyzed using real-time quantitative PCR and the 2(−Delta Delta C(T)) Method. The pig *β-actin* gene and GAPAH gene were used as reference genes for the normalization of the target gene data. The sequences of RT-qPCR primers are listed in Supplementary Table S1.

### 2.12. Statistical Analysis

Statistical analyses of the RT-qPCR results and graphs were carried out in GraphPad Prism (version 8.3) software (San Diego, CA, USA). The statistical significance of the data was tested by performing paired *t*-tests. The results are presented as means ± SEM of three replicates, and the statistical significance was represented as *p*-values < 0.05 or 0.01. Error bars show standard error of the mean, *** *p* < 0.001, ** *p* < 0.01, * *p* < 0.05.

## 3. Result

### 3.1. Overview of RNA Sequencing

The number of raw reads generated from each library exceeded 100 million reads. After filtering low-quality reads, each sample was sequenced to obtain at least 3G data, the clean reads ranged from 3.9G to 5.2G, and the clean read quality scores of Q20 and Q30 were above 94.72% and 88.46%, respectively. The GC content of the clean data ranged from 47.77% to 50.70% (Supplementary Table S2). More than 81.2% of the clean reads mapped to the genome using Hisat (v2.0.4) software (Supplementary Table S3). Therefore, the reliability and quality of the sequencing data were adequate for further analysis.

### 3.2. Features of mRNAs and lncRNAs

**Figure 1:**
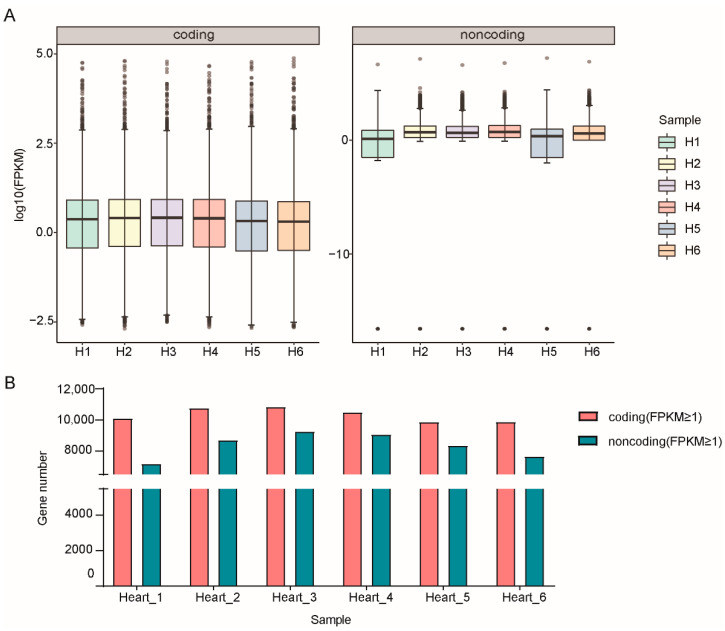
Statistics of all expressed genes identified from RNA-seq data in this study. (**A**) Expression levels of mRNAs and lncRNAs. (**B**) Gene numbers of mRNAs and lncRNAs with each sample.

The RPKM values of six samples in the two groups are shown in Supplementary Table S4. There were marked differences between coding and noncoding at the gene structure and expression levels. The distribution of RPKM values of lncRNAs was more concentrated, while that of mRNAs was more extensive. Moreover, the expression of mRNA was generally higher than the value of lncRNA in each sample (Figure 1A). After counting the numbers of mRNAs and lncRNAs in six samples, we found that the number of mRNAs with RPKM values ≥1 was significantly greater than those lncRNAs (Figure 1B). We also counted the ORF length and exon number of the coding and noncoding genes, finding that the ORF of mRNA was longer than noncoding and the exon number of mRNA was much more than the lncRNAs (Supplementary Figure S2).

**Figure 2:**
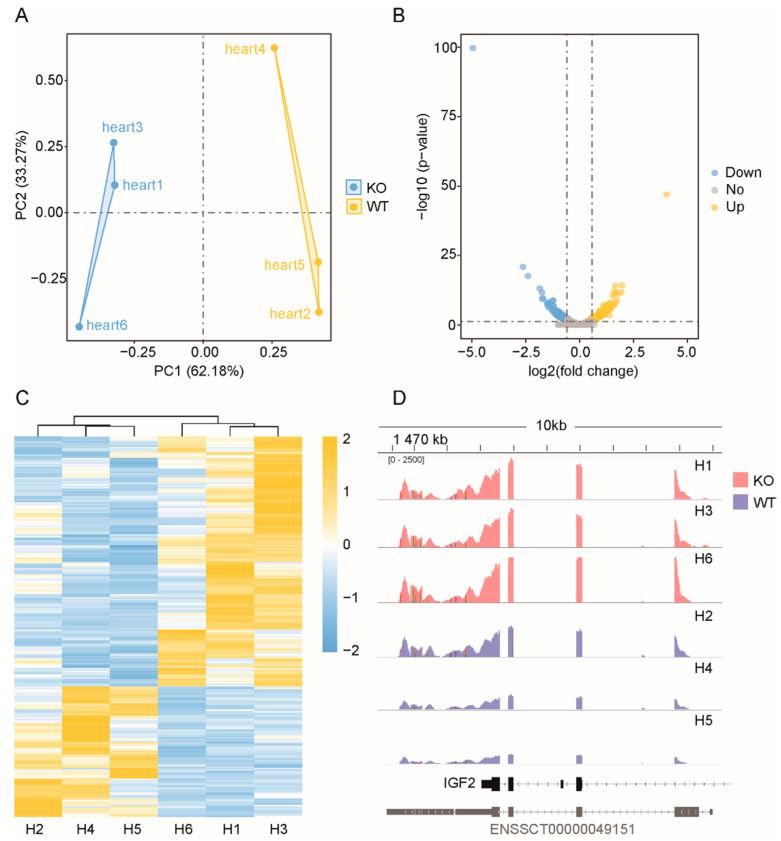
Statistical analysis of the coding genes. (**A**) PCA of all expressed mRNAs. Blue dots represent *ZBED6*-KO samples, whereas yellow dots represent *ZBED6*-WT. (**B**) Volcano plot of all expressed mRNAs. Blue represents down-regulated DEGs, yellow represents upregulated DEGs and gray represents insignificantly expressed genes. The horizonal dashed black line shows criteria of *p*-value equal to 0.05, while the vertical dashed lines show log2FC equal to −log1.5 (left) and log1.5 (right). (**C**) Hierarchical clustering of DEGs. The heavier the yellow is, the higher the difference in gene expression is, and the heavier the blue is, the lower the difference in gene expression is. (**D**) Direct comparison of the read counts (*y*-axis) from *ZBED6*-KO and *ZBED6*-WT RNA-seq data across IGF2 regions. The red represents *ZBED6*-KO group, and blue represents *ZBED6*-WT.

**Figure 3:**
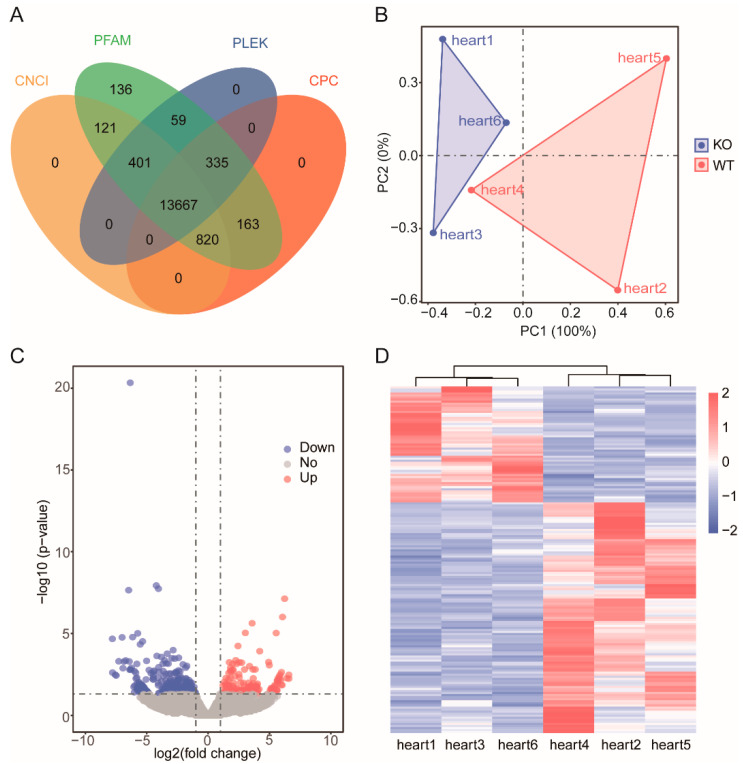
Statistical analysis of the noncoding genes. (**A**) Coding potentiality filter using Coding Potential Calculator (CPC), Pfam, PLEK, and Coding-Non-Coding Index (CNCI). (**B**) PCA of all expressed lncRNAs. Blue dots represent *ZBED6*-KO samples, whereas red dots represent *ZBED6*-WT. (**C**) Volcano plot of DELs. Blue represents down-regulated DEGs, and red represents upregulated DEGs. The horizonal dashed black line shows criteria of *p*-value equal to 0.05, while the vertical dashed lines show log2FC equal to −log1.5 (left) and log1.5 (right). (**D**) Hierarchical clustering of DEGs. The heavier the red is, the higher the difference in gene expression is, and the heavier the blue is, the lower the difference in gene expression is.

Principal component analysis of all genes and lncRNAs was conducted using the R language package. Figure 2A shows that the mRNAs expressed in the three samples of WT and the samples of the *ZBED6*-KO group were clustered together, with PC1 accounting for 62.18%, indicating that the repeatability of each group was good. Similarly, the PCA of lncRNAs showed the same result, which significantly separated the two groups, with PC1 accounting for 100% (Figure 3B). Although individual “Heart 4” deviated in the group, the cluster analysis of the differential genes deviated in the group, the cluster analysis of the differential genes indicated that the data were accurate and dependable and could satisfy the subsequent difference analysis.

### 3.3. Identification of lncRNAs

The protein-coding capability of all noncoding transcripts with CPC2, CNCI, Pfam and PLEK was predicted, and then the 13667 lncRNA was obtained by taking the intersection of four kinds of software results (Figure 3A).

After predicting the coding functions of the lncRNAs, the identification rate of the novel lncRNAs was shown in Supplementary Figure S3. It was divided into three categories, among which “Antisense lncRNA” accounted for 73.73%, “Inter lncRNA” accounted for 26.72% and “Intro lncRNA” accounted for 0%.

### 3.4. Differentially Expressed mRNAs and lncRNAs

According to the screening criteria, a total of 248 DEGs and 209 DELs were screened (Supplementary Table S5). The DEGs included 163 upregulated and 85 downregulated DEGs (Figure 2B). Among the 209 DELs, 66 were upregulated and 143 were downregulated in the *ZBED6*-KO group compared with the WT group, and the log2 ratio ranged from −6.3478 to 3.6507 (Figure 3C).

### 3.5. Hierarchical Cluster Analysis

Using ggplot2 in the R software package and the RPKM value of six samples to perform the hierarchical cluster analysis of the DEGs and DELs, the results, presented using a direct heatmap plot (Figure 2C and Figure 3D), showed that all gene expression patterns including mRNA and lncRNA patterns were more similar for three individuals (×1, ×3, ×6) in the *ZBED6*-KO group and for three individuals (×2, ×4, ×5) in the *ZBED6*-WT group. It was further demonstrated that the data obtained by RNA-seq were accurate and dependable.

### 3.6. Prediction of lncRNA Target Genes

To investigate the function of lncRNAs, we predicted the potential target genes of lncRNAs in cis actions by searching for protein-coding genes both 100 kb upstream and downstream of the lncRNAs. A total of 105 potential related cis-acting target genes were obtained (Supplementary Table S6), among which *ACTB*, *ACTC1*, *CDH15*, *HAND2*, *MYO16* and *MYOCD* genes are related to muscle growth and heart development and are located near *TCONS_00033689*, *TCONS_00091379*, *TCONS_00016689*, *TCONS_00021032*, *TCONS_00005021* and *TCONS_00068622*, respectively, thereby suggesting that the cis-acting genes and the neighboring lncRNAs could simultaneously play a role in growth and development of myocardium.

### 3.7. GO and KEGG Enrichment Analysis

**Figure 4:**
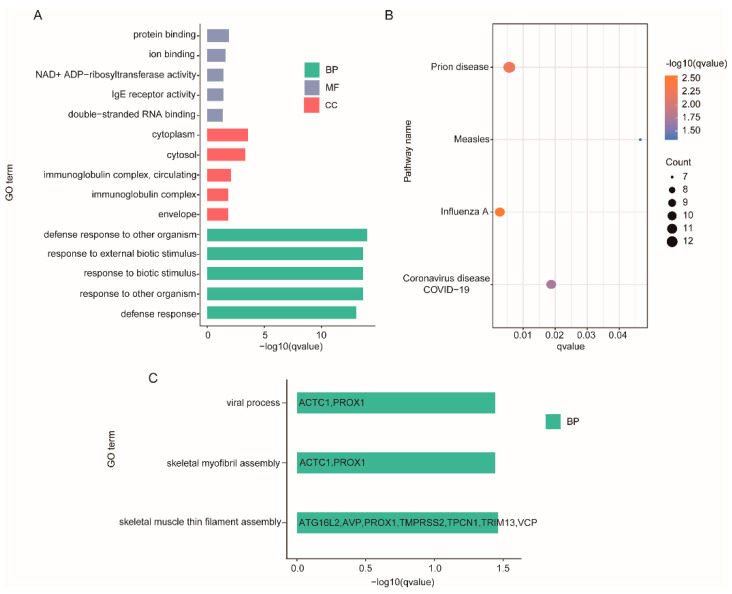
Enrichment analysis of DEGs and DELs. (**A**) The 15 most enriched GO terms in different classes of DEGs. (**B**) KEGG pathways of DEGs. (**C**) GO terms of DELs.

Using a *p*.adj ≤ 0.05 as the screening criteria, significant enrichment was found in 112 GO terms and 4 KEGG pathways from DEGs with 90 biological process, 10 cellular component terms and 12 molecular function as well as in 3 biological processes from cis target genes of DELs (Figure 4, Supplementary Table S7). Enrichment analysis of DEGs demonstrated that most of these genes were clustered into the immune process, such as three GO terms in different classes with lower *p*-values: defense response to other organisms, immunoglobulin complex, circulating and immunoglobulin receptor activity. Although no GO terms of DEGs enriched in muscle growth and development, there were several related genes, like *MYO19* and *TNNT1*. Among the GO terms enriched by cis target genes of DELs, there were two items related to muscle development, skeletal muscle thin filament assembly and skeletal myofibril assembly, and *ACTC1* was significantly enriched in these two terms. These findings demonstrate one of the roles of lncRNAs: the regulation of development through their cis actions on their neighboring protein-coding genes, e.g., regulating the *ACTC1* protein during muscle development.

### 3.8. ZBED6 Target Genes Analysis by ChIP-PCR

**Figure 5:**
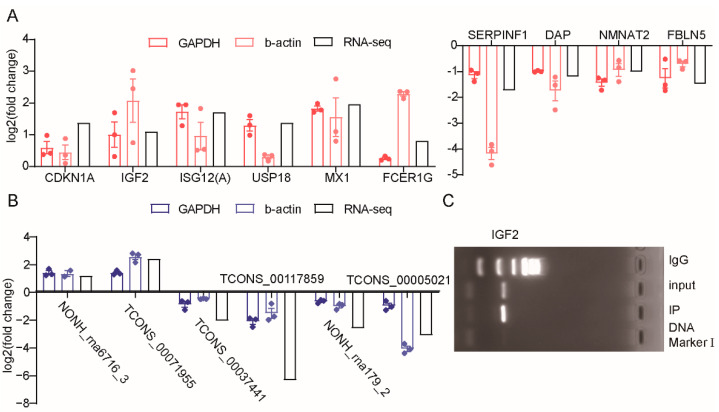
The verification of differentially expressed genes expression profiles. (**A**) Expression levels of six upregulated and four downregulated DEGs based on RNA-seq data and RT-qPCR. (**B**) Expression levels of two upregulated and three downregulated DELs based on RNA-seq data and RT-qPCR. Log2 fold change > 0 or <0 indicate the upregulated or downregulated in *ZBED6*-KO group compared to *ZBED6*-WT group. (**C**) The ChIP-PCR result of *IGF2* gene.

We examined the binding of *IGF2* and *ZBED6* to verify the DNA in ChIP experiments. The blast rate of IGF2 binding sequence in C2C12 with *Sus scrofa IGF2* located on chromosome 2 was as high as 86%, and the ChIP-PCR result showed that *IGF2* could bind with *ZBED6* in the myocardium, which indicated that the DNA by ChIP was reliable and usable (Figure 5C).

### 3.9. Verification of Gene Expression Profiles with RT–qPCR

To confirm the expression patterns of the mRNA and lncRNA, ten DEGs and six DELs were selected for RT–qPCR validation. We selected *b-actin* and *GAPDH* as the reference genes and assumed that the expression of the reference genes was constant for all the samples. The DEG expression levels were determined and compared with the RNA-seq data, which showed similar patterns to the RNA-seq data (Figure 5A), and five DELs provided the same result (Figure 5B).

To investigate the expression of candidate genes in offspring, we also quantitatively verified *IGF2* and *TCONS_00005021* in the myocardium tissue of F3 generation pigs. The result showed that the expression of *IGF2* was significantly upregulated and the *TCONS_00005021* was downregulated after ZBED6 knockout with *GAPDH* as reference gene (Figure S4).

**Figure 6:**
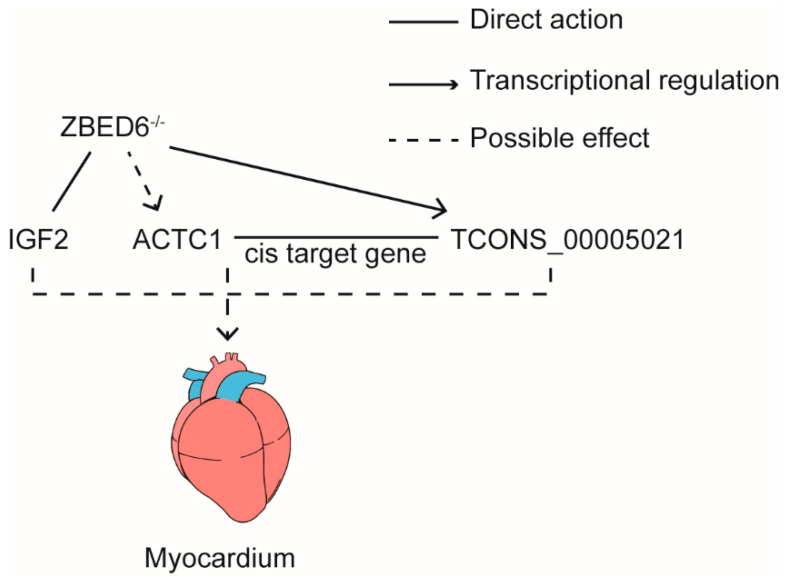
Working model: *ZBED6* could directly influence the expression of IGF2 and *TCONS_00005021.* Also, *TCONS_00005021* regulates the expression of *ACTC1* by cis-mode.

Based on the above results, we hypothesized a working model in which *ZBED6*-IGF2 axis was the major influence on myocardial development by direct interaction with *IGF2*. Concurrently, *ZBED6* also regulated the expression of *TCONS_00005021* that regulate the expression of *ACTC1* in cis-mode. It was another factor to promote the growth of the myocardium (Figure 6).

## 4. Discussion

ZBED6 can inhibit the expression of *IGF2*, reduce the deposition of subcutaneous fat, promote the growth of skeletal muscle [26] and also affect the growth and development of the myocardium [40]. By knocking in the same *IGF2* intron 3–3072 site, *ZBED6* improved meat production in Chinese Bama Xiang pigs [41]. We identified the core lncRNAs and mRNAs by performing comprehensive analysis of RNA-seq data from *ZBED6* knockout and wild-type Bama Xiang pigs and validated their expression with RT-qPCR. Overall, our work uncovered some candidate genes that are involved in myocardial development in *ZBED6*-KO pigs.

By comparing the characteristics of mRNA and lncRNA, we found that the expression and exon number of lncRNA were less than that of mRNA, which was consistent with the results of Lagarde et al. [42]. In addition, after screening and statistics with “value one” as the threshold, we found that the gene number of lncRNA in six individuals was less than that of mRNA. This was because the low expression of lncRNA and the proportion of lncRNA in mammalian cells was significantly less than that of mRNA [43].

In this paper, 248 DEGs and 209 DELs were screened by transcriptome analysis, which should have a close relationship with the knocked-out *ZBED6* gene. After *ZBED6* knockout, the IGV of RNA-seq results showed that *IGF2* was significantly upregulated in myocardial tissue. Previous studies have confirmed with RT–qPCR that *IGF2* expression is significantly higher in *ZBED6*-KO group than in the *ZBED6*-WT group [26], which is consistent with the results in mice and cattle [4,6,7]. We again confirmed the reliability of our data.

As the first organ formed in the process of vertebrate growth and development, the heart is mostly composed of myocardial tissue. Previous histological analysis of myocardial HE-stained sections showed that knockout of *ZBED6* resulted in thicker myocardial muscle fibers and a reduction in connective tissue. These findings are complementary to the heart weight results, indicating that *ZBED6* knockout could promote the growth and development of pig hearts to a certain extent [26].

In the enrichment analysis of cis target genes of DELs, we found that there were two items, skeletal myofibril assembly and skeletal muscle thin filament assembly, related to muscle development, and *ACTC1* was significantly enriched in these two GO terms. *ACTC1* (actin α cardiac muscle1) is an α-actin protein found in cardiac muscle and is the main component of thin-filament genes in mature cardiac myocytes, and it is completely conserved in mammals. Mutations in *ACTC1* have been phenotypically related to various cardiac anomalies including dilated cardiomyopathy (DCM), restrictive cardiomyopathy (RCM), left ventricular non-compaction (LVNC), HCM, and congenital heart disease (CHD) [44,45,46]. Mice lacking *ACTC1* do not survive more than two weeks, and knockdown of *ACTC1* in chick embryos causes ASD [44,47]. Findings confirmed that *ACTC1* is critical for normal cardiac morphogenesis and muscle contraction [48]. Based on our results, we believe that the expression of *TCONS_00005021* was significantly downregulated after *ZBED6* knockout and that this affects the expression of its cis target gene *ACTC1*. Although *ACTC1* is not a differentially expressed gene, it changes its original transcriptional expression due to the regulation of lncRNA, which has a certain effect on the myocardium of Bama Xiang pig.

Although there were no significantly enriched GO terms or pathways of DEGs related to muscle or heart development, *TNNT1* and *MYO19* were significantly expressed after *ZBED6* knockout.

The coordination of the TPM (tropomyosin)–TNN (troponin)–actin complex and myosin controls muscle contraction and relaxation [49,50]. TNNT (mammalian troponin T), TNNI and TNNC are troponin members. These proteins are important for regulating the contraction of myofibrils and maintaining the structural integrity of the myofibril ganglion [51]. Three homologous genes have evolved in vertebrates to encode three muscle type-specific TnT isoforms: *TNNT1* for slow skeletal muscle TnT. Troponin T (TnT) is a central player in the calcium regulation of actin thin filament function and is essential for the contraction of striated muscles. Mutations in *TNNT1* resulting in a complete loss of slow TnT in slow skeletal muscle also causes severe nemaline myopathy with childhood lethality [52,53]. The alternative splicing regulation of *TNNT1* expression may play an important role in modulating muscle contractility in physiological and pathophysiological adaptations. Typical histological features of TNNT1 NM (Nemaline) include rods in type I and II fibers, increased fiber size variation, fiber type disproportion and selective type II fiber hypertrophy [54,55]. We think *TNNT1* could play a certain role in muscle contraction: after *ZBED6* knockout, the expression of *TNNT1* was significantly upregulated and then promoted muscle contraction, thus affecting the growth and development of the myocardium.

Myosins are actin-based motors that power processes such as muscle contraction, cytokinesis and organelle transport [56]. Myosin-XIX (Myo19), a novel myosin, functions as an actin-based motor for mitochondrial movement in vertebrate cells [57]. *MYO19* is broadly expressed in vertebrate cells, tissues and tumors. However, the mechanism of actin-based mitochondrial movements in vertebrates remains unclear [58]. The downregulation of *MYO19* expression mediated by ZBED6 may provide a theoretical basis for the myocardial development and movement mechanism of actin in mitochondria.

ZBED6 could inhibit the expression of *IGF2* through the combination of “GCTCG” motif. Using ChIP-PCR experiment, we first demonstrated that ZBED6 could bind with *IGF2* in myocardial tissue through this binding motif, and we proved this point again in skeletal muscle and liver tissues of *ZBED6*-KO pigs [26], which is consistent with previous research results in long-white pigs and mice [5,59]. These findings indicated that in the myocardial tissue of Bama Xiang pig, the ZBED6-IGF2 axis still plays an important role in the growth and development of the myocardium. IGF2, as the main target of ZBED6, not only plays a role in skeletal muscle tissue but also has a significant effect on myocardial tissue.

Therefore, we believe that knockout of *ZBED6* increases heart weight by promoting the growth and development of the myocardium. At present, porcine heart has been successfully transplanted into baboon [60], and the studies have found the structural characteristics of the porcine heart valve [61]. In this study, we clarified the transcriptional expression profile in the myocardium of Bama Xiang pig, which could provide a theoretical basis and practical model for the study of heart organ transplantation and cardiovascular disease in pigs.

## 5. Conclusions

Through high-throughput RNA-seq, a total of 248 differentially expressed genes (DEGs) and 209 differentially expressed lncRNAs (DELs) were obtained between *ZBED6*-KO and *ZBED6*-WT pigs. The *TNNT1* and *MYO19* were both significantly expressed between groups, which had a certain effect on the development of muscle and myocardium. Based on the target gene prediction of DELs, a total of 105 potential related cis target genes were found. The DEGs were significantly enriched in 112 GO terms and 4 KEGG pathways. Additionally, there was significant enrichment in three GO terms of the cis target genes of DELs, among which two GO terms were related to muscle growth and development, and *ACTC1* was significantly enriched in both two items. *IGF2* was confirmed to be the target gene of ZBED6 in Bama Xiang pig by direct binding in ChIP-PCR experiments. We regard *IGF2*, *TCONS_00005021* and its target gene-*ACTC1* as key candidate genes in promoting myocardial growth and development in Bama Xiang pigs after *ZBED6* gene knockout.

## Data Availability

All the RNA-seq reads have been deposited in the Sequence Read Archive (https://www.ncbi.nlm.nih.gov/sra accessed on 1 June 2021) with accession codes (BioProject ID: PRJNA663759).

## Supplementary Materials

The following supporting information can be downloaded at: https://www.mdpi.com/article/10.3390/genes13081382/s1, Figure S1: Overview of the experiment design, Figure S2: Features of mRNA and lncRNA, Figure S3: Classification of the novel lncRNAs, Figure S4: Verification of candidate genes in F3 generation pigs. Table S1: A list of primers, Table S2: The quality analysis of sequencing data, Table S3: The genome mapping of sequencing data, Table S4: A expression of all lncRNAs, Table S5: A list of all differentially expressed genes, Table S6: Potential related genes of differentially expressed lncRNA, Table S7: A enrichment analysis of DEGs.
