# Supplementary material for: A Comprehensive Analysis of the Myocardial Transcriptome in ZBED6-Knockout Bama Xiang Pigs
Source: Genes (Basel). 2022 Aug 1;13(8):1382. doi: 10.3390/genes13081382 (PMC9407500; doi:10.3390/genes13081382)
Supplement: Supplementary file 1 [file genes-13-01382-s001.zip › Supplementary Materials.pdf]

Supplementary Materials

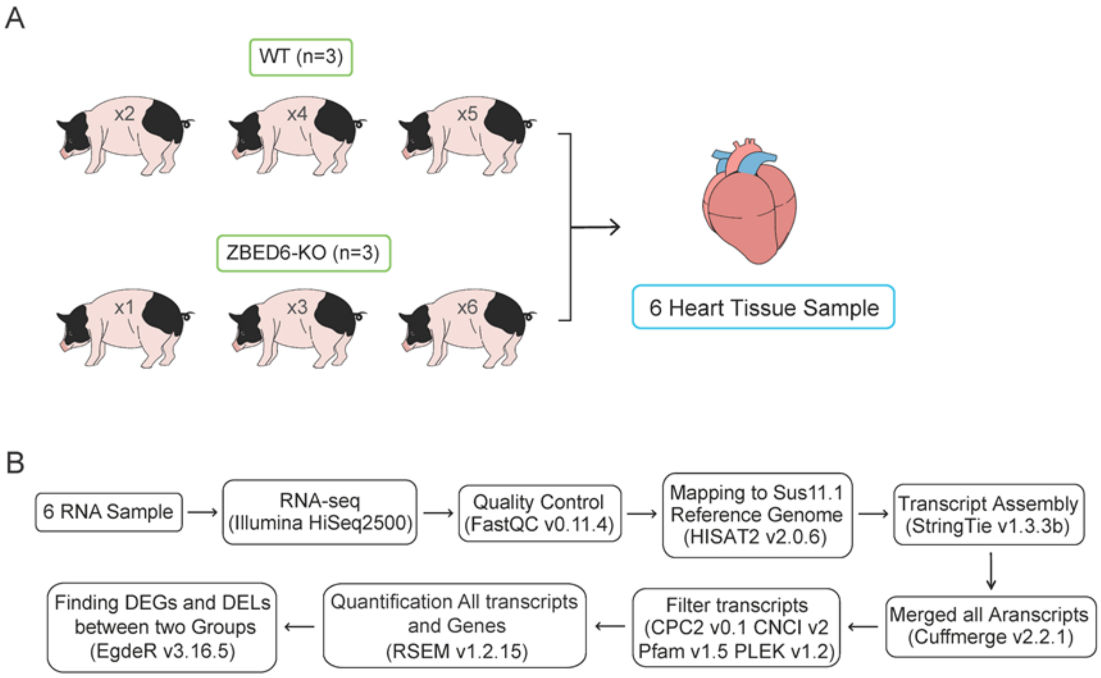

Supplementary Figure S1. Overview of the experiment design. (A) Total of six healthy Bama Xiang pigs were selected in this study. The heart tissue sample was collected from *ZBED6*-WT and *ZBED6*-KO groups after slaughtering. (B) RNA-seq sequencing flow chart and data analysis.

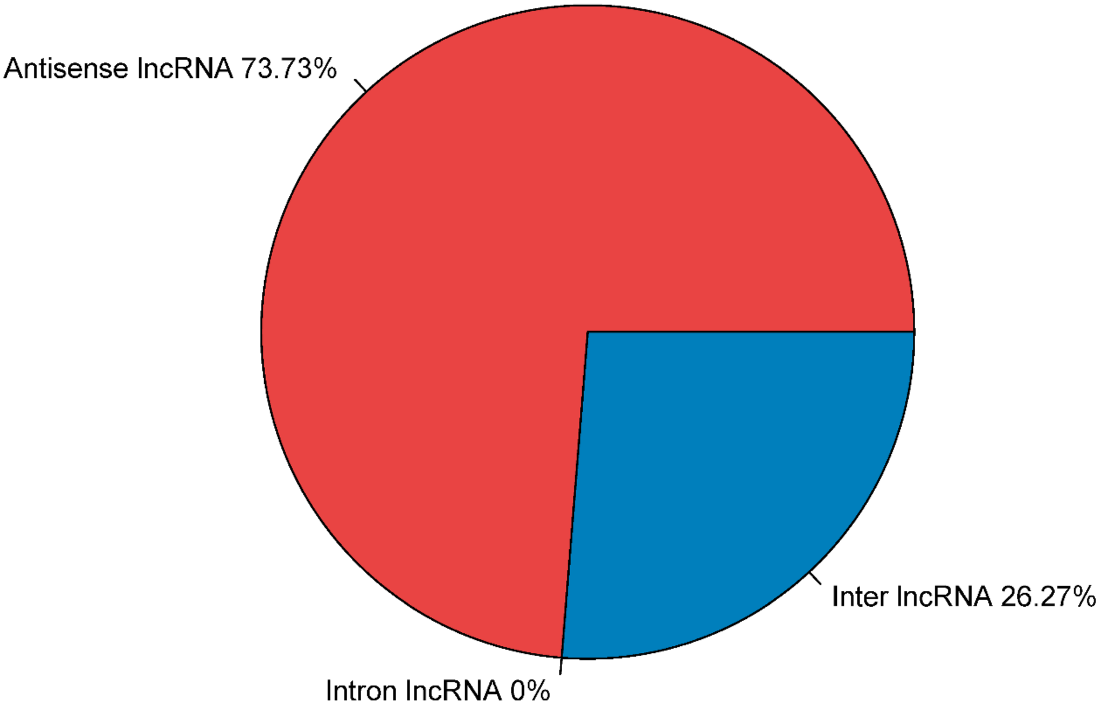

Supplementary Figure S2. Features of mRNA and lncRNA. (A) The ORF length of mRNA and lncRNA. (B) The exon number of mRNA and lncRNA.

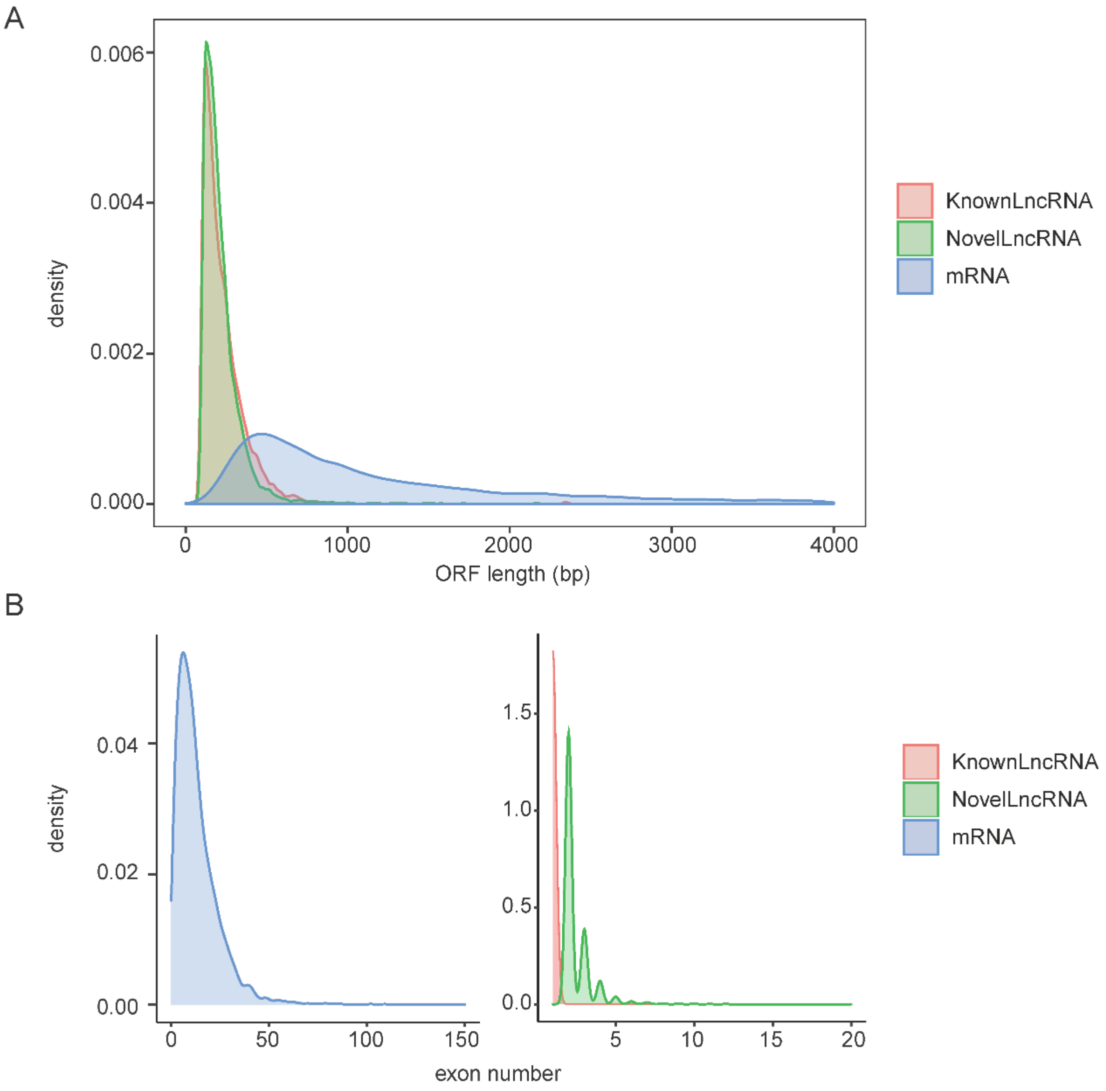

Supplementary Figure3. Classification of the novel lncRNAs.

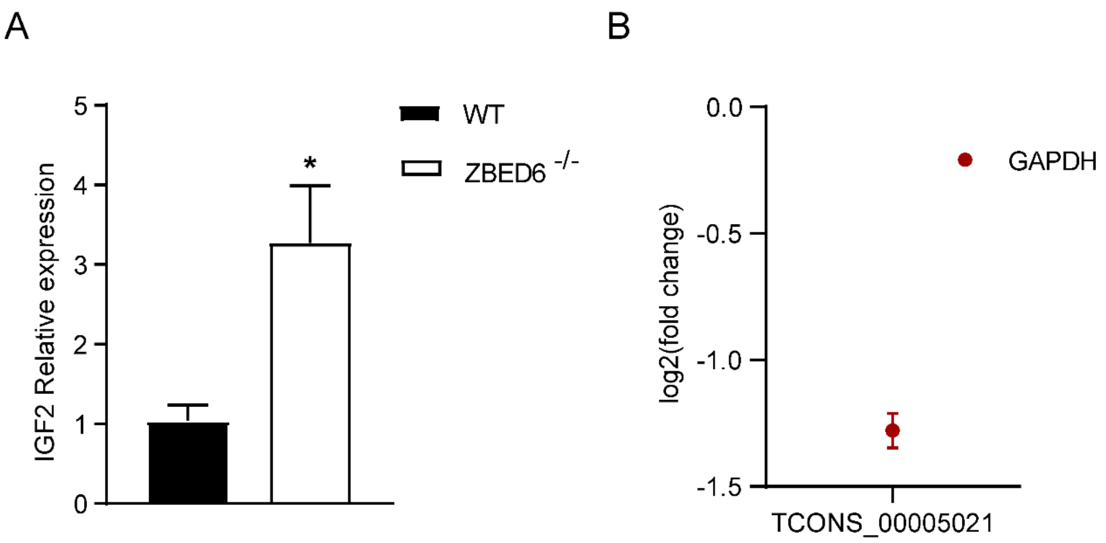

Supplementary Figure4. Verification of candidate genes in F3 generation pigs. (A) The RT-qPCR of *IGF2* gene. (B). Expression level of *TCONS_00005021* based on RT-qPCR with the *GAPDH* as

reference.
